# Supplementary material for: Chromothripsis during telomere crisis is independent of NHEJ, and consistent with a replicative origin
Source: Genome Res. 2019 May;29(5):737–49. doi: 10.1101/gr.240705.118 (PMC6499312; doi:10.1101/gr.240705.118)
Supplement: Supplemental Material [file supp_gr.240705.118_Supplemental_file_1.zip › contigs/annotated_contigs/DB76/contig.2.DB76_length_616_mean_cov_8.66558441558.docx]

**DB76_length_616_mean_cov_8.66558441558**

```
GCCTTCCTGGGTGATGACCTGGCGGCAGATCCCACAGGGCTTCACCTTCTTGAAGGTCTTCACCTTGAAGCGGTGTGTCTTGGGGGCCT
 >chr2:218855677-218856057 + E=5e-217
CCAGATCCTCTGGCTGTGGGAGGAGAGGCACAGAGTCAGCCCTGGGTAGAAGGCTGGGGTCCTGGGGAAGGAGTAGAGGAGCCTGGGGG

AGAGGTAGGCAGGGACAGGGAAAAGGATGGCAAGAGCCCCTCAGGGTCCAGAGGAGGGAGGAGGGCCCAGGGACAGAGAAAGGTGTGGG

GTGACAGGATGTTCATCCCAGACTCAGGTGGGGCCCATGGCCTGAGTCCAGACGGCAGCTGGAGAGTCCAGCTCCAGGGACCTGCAGCC

CCCGCATGGCTCCTACCTTGG|CTT|CTAGTCCCCACCATTGCTGCTATTGCAGTGTGCCTGGGACCCTTAGAGCCCCACAGTATCTCC
                      >chr2:218650148-218650387 + E=4e-132
GGAGAAAGCCATCCCTCTGCCTCCTGTGTCCCCGGCTCAGGGACATCGCTCACCAAGGGAGCCTGATACGTGAAAGAAGTTCAAATGTT

CTATCAACTGGGAAGGCCTGCGGCAGCAATGGGCACTTCCTCTGCACCACAGCCATCCGACCACAAGAGGGCGACATCCCCCTT
```
